# An unbiased test reveals no enrichment of sexually antagonistic polymorphisms on the human X chromosome

**DOI:** 10.1098/rspb.2021.2314

**Published:** 2022-01-26

**Authors:** Filip Ruzicka, Tim Connallon

**Affiliations:** School of Biological Sciences, Monash University, Clayton, Victoria, Australia

**Keywords:** sexually antagonistic selection, theoretical models, humans, sex chromosomes, empirical population genomics, *F*_ST_

## Abstract

Mutations with beneficial effects in one sex can have deleterious effects in the other. Such ‘sexually antagonistic’ (SA) variants contribute to variation in life-history traits and overall fitness, yet their genomic distribution is poorly resolved. Theory predicts that SA variants could be enriched on the X chromosome or autosomes, yet current empirical tests face two formidable challenges: (i) identifying SA selection in genomic data is difficult; and (ii) metrics of SA variation show persistent biases towards the X, even when SA variants are randomly distributed across the genome. Here, we present an unbiased test of the theory that SA variants are enriched on the X. We first develop models for reproductive *F*_ST_—a metric for quantifying sex-differential (including SA) effects of genetic variants on lifetime reproductive success—that control for X-linked biases. Comparing data from approximately 250 000 UK Biobank individuals to our models, we find *F*_ST_ elevations consistent with both X-linked and autosomal SA polymorphisms affecting reproductive success in humans. However, the extent of *F*_ST_ elevations does not differ from a model in which SA polymorphisms are randomly distributed across the genome. We argue that the polygenic nature of SA variation, along with sex asymmetries in SA effects, might render X-linked enrichment of SA polymorphisms unlikely.

## 1. Introduction

Adaptation requires genetic variation for fitness, yet we know surprisingly little about its genetic basis [1,2]. Is fitness variation mostly attributable to rare variants maintained by recurrent mutation, or to common variants maintained by various forms of balancing selection? To what extent are the frequencies of fitness-affecting variants influenced by genetic drift, departures from equilibrium or gene flow from neighbouring populations? To what extent do loci with large versus small fitness effects contribute to genome-wide fitness variation? Also, how are these genetic variants distributed across the genome? These are some of the most pressing questions in evolutionary biology, yet they are also the most challenging to answer [3].

Sexually antagonistic (SA) polymorphisms, wherein the alleles of a locus have opposing fitness effects on each sex, are an important class of fitness-affecting genetic variant. The basic conditions giving rise to SA fitness effects—including sex differences in selection on traits expressed by both sexes, and sex-differential phenotypic effects of mutations—are permissive [4] and have been widely documented in natural and experimental populations [5–8]. Accordingly, direct evidence for SA genetic variation has been reported in numerous quantitative genetic studies (e.g. [9–11]; see also [12]). Yet despite this, our understanding of the genomic distribution, fitness effects and evolutionary dynamics of SA variants remains poor [13–16].

An intriguing and oft-debated question is whether SA polymorphisms are more likely to be found on autosomes or the X chromosome. This question has inspired an extensive body of theoretical research (e.g. [17–23]) that has identified conditions where the X chromosome should be enriched, or deficient, for SA polymorphisms. Rice [18], for example, highlighted conditions under which the X is a more permissive genomic location for balancing selection of SA alleles, while Fry [20] later emphasized the sensitivity of Rice's predictions to the dominance relations between SA alleles, about which little is known [24]. Moreover, when SA polymorphisms are maintained by recurrent mutation rather than balancing selection, the X chromosome should typically harbour fewer SA polymorphisms, owing to stronger purifying selection and elevated rates of genetic drift at X-linked compared to autosomal loci [25–28]. In short, empirical studies—rather than theory alone—are needed to resolve whether the X, autosomes, or neither chromosome type, are enriched for SA polymorphisms.

So far, two formidable challenges have hampered empirical research on this question. The first is the logistical difficulty of identifying SA polymorphisms using current genomic approaches (e.g. [29–33]), which typically lack the statistical power needed to confidently identify SA loci [16,29,34,35]. The second is that X-linkage inflates empirical signals of SA polymorphism, which can lead to biases in detection and erroneous inferences of elevated SA polymorphism on the X [23]. These biases arise, on the one hand, from less constrained conditions for allele frequency divergence between the sexes at X-linked compared to autosomal loci [23,29,30], and, on the other hand, from the inherently stronger effects of X-linked compared to autosomal polymorphisms on male fitness variances and cross-sex fitness covariances [23,28,36–38]. These twin challenges of estimation and inference render previous tests for an enrichment of SA polymorphisms on the X chromosome ambiguous [30,39–47].

Here, we develop and implement an unbiased test for the enrichment of SA polymorphisms on the X chromosome. Building on recently developed methods for characterizing polygenic signals of SA polymorphism in population genomic data [48], we first test for polygenic signals of SA polymorphism on the X chromosome and autosomes, using genotypic and lifetime reproductive success (LRS) data from approximately 250 000 males and females from the UK Biobank [49]. Our metric of SA polymorphism, ‘reproductive *F*_ST_’, quantifies sex differences in the genetic basis of LRS among UK Biobank adults, and thus controls for X-autosome biases in between-sex allele frequency differences that may arise between fertilization and reproductive maturity [23,29,30]. We then develop an idealized model of reproductive *F*_ST_ in which the prevalence and attributes of SA polymorphisms do not systematically differ between the X and autosomes. By comparing polygenic signals of SA polymorphism in the UK Biobank to this theoretical baseline, we control for both the elevated sampling variance and stronger effects of X-linked polymorphisms on the reproductive *F*_ST_ metric [23,28,36–38], allowing us to assess whether SA polymorphisms are disproportionately autosomal or X-linked.

## 2. Theoretical background

Our empirical analyses address two questions. First, is there a polygenic signal of SA polymorphism on the human X chromosome in the UK Biobank dataset (an autosomal signal was previously reported by Ruzicka *et al*. [48])? Second, do the magnitudes of these signals imply an elevation of SA polymorphisms on the X relative to autosomes? To formally test each question, we developed mathematical models for ‘reproductive *F*_ST_’—a metric that potentially captures sex-differential (including SA) effects of alleles on LRS (see below and [48])—and compared empirical data to each model. For the first question, we developed null models for the distribution of reproductive *F*_ST_ *in the absence* of sex differences in selection. For the second question, we developed models of reproductive *F*_ST_ in which SA polymorphisms are present and inflate reproductive *F*_ST_ on both the X and autosomes, yet neither chromosome is enriched for them (i.e. there are no systematic differences between chromosomes in the abundance or attributes of SA polymorphisms). This second model accounts for other factors that are likely to differ between the X and autosomes, including the effects of diploidy versus haploidy on the expression of fitness variation. We elaborate on each model of reproductive *F*_ST_ below.

### (a) Definition of reproductive *F*_ST_

Our models and empirical analyses focus on bi-allelic loci (i.e. the vast majority of polymorphic sites in population genomic datasets). For an X-linked locus with alleles *A*_1_ and *A*_2_, females have three genotypes (*A*_1_*A*_1_, *A*_1_*A*_2_ and *A*_2_*A*_2_) and males have two (*A*_1_ and *A*_2_). Let *n*_11,*f*_, *n*_12,*f*_ and *n*_22,*f*_ represent the number of females of each genotype recorded in the UK Biobank dataset, respectively (*N_f_* = *n*_11,*f*_ + *n*_12,*f*_ + *n*_22,*f*_). The frequency of the *A*_1_ allele in the female sample is ${\hat{\;p}}_{f}=(n_{11,f}+(1/2)n_{12,f})N_{f}^{-1}$. Likewise, *n*_1,*m*_ and *n*_2,*m*_ represent the number of males from the UK Biobank that carry alleles *A*_1_ and *A*_2_ (*N_m_* = *n*_1,*m*_ + *n*_2,*m*_), and ${\hat{\;p}}_{m}=n_{1,m}N_{m}^{-1}$ is the *A*_1_ allele frequency in the male sample.

Letting *F_ij_* represent the total number of offspring produced by females carrying genotype *ij* (*ij* = {11, 12, 22}), and *M_i_* represent the total number of offspring produced by males carrying genotype *i* (*i* = {1, 2}), the expected frequencies with which females and males transmit the *A*_1_ allele to their offspring (respectively) are

2.1 $$\begin{array}{rl} {{\hat{\;p}}^{\prime }}_{f}= & \frac{F_{11}+(1/2)F_{12}}{F_{11}+F_{12}+F_{22}}\\ \mathrm{and}\;{{\hat{\;p}}^{\prime }}_{m}= & \frac{M_{1}}{M_{1}+M_{2}}. \end{array}\}$$

Reproductive *F*_ST_ is a standardized measure of sex differences in allele transmission, owing to sex-specific genetic variation for LRS, and controlling for allele frequency differences between adults [48]. It is defined as

2.2 $${\hat{F}}_{\mathrm{ST}}=\frac{{\left(\left({{\hat{\;p}}^{\prime }}_{f}-{{\hat{\;p}}^{\prime }}_{m})-({\hat{\;p}}_{f}-{\hat{\;p}}_{m}\right)\right)}^{2}}{4\hat{\;p}(1-\hat{\;p})},$$

where $\hat{\;p}=({\hat{\;p}}_{f}+{\hat{\;p}}_{m})/2$. The same expression applies to autosomal loci, once male allele frequencies are adjusted to include diploidy in males, i.e.: ${\hat{\;p}}_{m}=(n_{11,m}+(1/2)n_{12,m})N_{m}^{-1}\;$ and ${\hat{\;p}}_{m}^{\prime }=(M_{11}+(1/2)M_{12}){\left(M_{11}+M_{12}+M_{22}\right)}^{-1}$.

### (b) Reproductive *F*_ST_ in the absence of sex differences in selection

With large sample sizes (as in the UK Biobank), no sex differences in selection and excluding polymorphic loci in which the minor allele frequency (MAF) is very low (i.e. less than 1% in the UK Biobank; see below), reproductive *F*_ST_ estimates are well-approximated by a *χ*^2^ distribution, with estimates for an X-linked and autosomal locus, respectively, as

2.3a $${\hat{F}}_{ST}\approx \frac{{\hat{p}}_{f}(1-{\hat{p}}_{f})(1-{\hat{F}}_{IS}^{f})\sigma_{f}^{2}/(2N_{f}\mu_{f}^{2})+{\hat{p}}_{m}(1-{\hat{p}}_{m})\sigma_{m}^{2}/(N_{m}\mu_{m}^{2})}{4\hat{p}(1-\hat{p})}X$$

and

2.3b $${\hat{F}}_{ST}\approx \frac{{\hat{p}}_{f}(1-{\hat{p}}_{f})(1-{\hat{F}}_{IS}^{f})\sigma_{f}^{2}/(2N_{f}\mu_{f}^{2})+{\hat{p}}_{m}(1-{\hat{p}}_{m})(1-{\hat{F}}_{IS}^{m})\sigma_{m}^{2}/(2N_{m}\mu_{m}^{2})}{4\hat{p}(1-\hat{p})}X$$

where *X* is a *χ*^2^ random variable with one degree of freedom, *μ_f_*, *μ_m_*, $\sigma_{f}^{2}$ and $\;\sigma_{m}^{2}$ correspond to the means (*μ_f_*, *μ_m_*) and variances ($\sigma_{f}^{2}$, $\;\sigma_{m}^{2}$) for female and male LRS (respectively) in the UK Biobank, ${\hat{F}}_{\mathrm{IS}}^{f}$ is a measure of the deviation of female genotype frequencies from Hardy–Weinberg expectations (we define ${\hat{F}}_{\mathrm{IS}}$ as in [16,34], where ${\hat{F}}_{\mathrm{IS}}>0$ when heterozygotes are in excess of Hardy–Weinberg predictions, and ${\hat{F}}_{\mathrm{IS}}<0$ when heterozygotes are deficient; see the electronic supplementary material); ${\hat{F}}_{\mathrm{IS}}^{m}$ is the deviation in the male sample. With genotype frequencies near Hardy–Weinberg expectations and ${\hat{\;p}}_{f}\approx {\hat{\;p}}_{m}$, these expressions simplify—for X-linked and autosomal loci, respectively—to

2.4a $${\hat{F}}_{\mathrm{ST}}\approx \left(\frac{1}{8N_{f}}\frac{\sigma_{f}^{2}}{\mu_{f}^{2}}+\frac{1}{4N_{m}}\frac{\sigma_{m}^{2}}{\mu_{m}^{2}}\right)X$$

and

2.4b $${\hat{F}}_{\mathrm{ST}}\approx \left(\frac{1}{8N_{f}}\frac{\sigma_{f}^{2}}{\mu_{f}^{2}}+\frac{1}{8N_{m}}\frac{\sigma_{m}^{2}}{\mu_{m}^{2}}\right)X.$$

### (c) Reproductive *F*_ST_ assuming a random genomic distribution of sexually antagonistic polymorphisms

To test whether SA polymorphisms are enriched on the X chromosome, we must first define a model for reproductive ${\hat{F}}_{\mathrm{ST}}$ in which SA polymorphisms are present, randomly distributed across the genome, and where the frequencies and fitness effects of SA polymorphisms do not systematically differ between the X and autosomes. We expect that there will be many specific evolutionary genetic scenarios that can lead to equivalent patterns of SA polymorphism between autosomes and the X. For example, when SA polymorphisms are maintained at equilibrium under balancing selection, there are specific conditions of dominance between SA alleles that generate identical equilibrium frequencies of balanced SA alleles between the X and autosomes [23]. More complicated contexts of selection, including interactions between recurrent mutation, genetic drift and the distributions of sex-specific selection and dominance coefficients, may also sometimes result in similar patterns of SA polymorphism on the X and autosomes (although we expect that levels of polymorphism maintained by recurrent mutation will tend to be higher on autosomes, given the enhanced purifying selection and genetic drift that have been predicted [25] and documented (e.g. [28,50] for X-linked genes). In our baseline model of equivalence between the X and autosomes, we, therefore, focus on the case where SA polymorphisms are maintained at equilibrium under balancing selection, which allows us to set up a testable prediction regarding the signal of SA polymorphism on the X versus the autosomes. Whether this idealized scenario is a good model for SA polymorphism in the genomes of humans or other species is a point we return to in the Discussion.

For simplicity, we consider the case where adult genotype frequencies are approximately equal between the sexes and close to Hardy–Weinberg expectations, as is largely the case within the UK Biobank dataset. For a set of *n_X_* and *n_A_* polymorphic SA loci on the X chromosome and autosomes, respectively, the expected *inflation* of mean ${\hat{F}}_{\mathrm{ST}}$ (relative to null expectations defined in equations (2.4*a,b*)) is

2.5a $$f_{X}^{\mathrm{sel}}=\bar{{\hat{F}}_{\mathrm{ST},X}}-\frac{1}{8N_{f}}\frac{\sigma_{f}^{2}}{\mu_{f}^{2}}-\frac{1}{4N_{m}}\frac{\sigma_{m}^{2}}{\mu_{m}^{2}}=\frac{1}{n_{X}}\overset{n_{X}}{\underset{i=1}{\sum }}\frac{{\hat{\;p}}_{i}(1-{\hat{\;p}}_{i})}{4}{\left[\frac{1}{2{\bar{w}}_{\;f,i}}\frac{d{\bar{w}}_{\;f,i}}{d{\hat{\;p}}_{i}}-\frac{1}{{\bar{w}}_{m,i}}\frac{d{\bar{w}}_{m,i}}{d{\hat{\;p}}_{i}}\right]}^{2}$$

and

2.5b $$f_{A}^{\mathrm{sel}}=\bar{{\hat{F}}_{\mathrm{ST},A}}-\frac{1}{8N_{f}}\frac{\sigma_{f}^{2}}{\mu_{f}^{2}}-\frac{1}{8N_{m}}\frac{\sigma_{m}^{2}}{\mu_{m}^{2}}=\frac{1}{n_{A}}\overset{n_{A}}{\underset{i=1}{\sum }}\frac{{\hat{\;p}}_{i}(1-{\hat{\;p}}_{i})}{4}{\left[\frac{1}{2{\bar{w}}_{\;f,i}}\frac{d{\bar{w}}_{\;f,i}}{d{\hat{\;p}}_{i}}-\frac{1}{2{\bar{w}}_{m,i}}\frac{d{\bar{w}}_{m,i}}{d{\hat{\;p}}_{i}}\right]}^{2},$$

where $\bar{{\hat{F}}_{\mathrm{ST},X}}$ and $\bar{{\hat{F}}_{\mathrm{ST},A}}$ denote mean ${\hat{F}}_{\mathrm{ST}}$ among these loci (see the electronic supplementary material). The terms in square brackets capture effects of sex-differential selection on ${\hat{F}}_{\mathrm{ST}}$. Note that when there are no sex differences in selection (i.e. the terms in square brackets evaluate to zero), there will be no inflation (i.e. $f_{X}^{\mathrm{sel}}=0$ and $f_{A}^{\mathrm{sel}}=0$), and $\bar{{\hat{F}}_{\mathrm{ST},X}}$ and $\bar{{\hat{F}}_{\mathrm{ST},A}}$ conform to the null models in equation (2.4).

When fitness effects of SA alleles are small [19,51], single-generation allele frequency changes at X-linked and autosomal loci (respectively) are well-approximated by

2.6a $$\Delta p_{X,i}=\frac{\;p_{i}(1-p_{i})}{3}\left[\frac{1}{{\bar{w}}_{\;f,i}}\frac{d{\bar{w}}_{\;f,i}}{dp_{i}}+\frac{1}{{\bar{w}}_{m,i}}\frac{d{\bar{w}}_{m,i}}{dp_{i}}\right]$$

and

2.6b $$\Delta p_{A,i}=\frac{\;p_{i}(1-p_{i})}{4}\left[\frac{1}{{\bar{w}}_{\;f,i}}\frac{d{\bar{w}}_{\;f,i}}{dp_{i}}+\frac{1}{{\bar{w}}_{m,i}}\frac{d{\bar{w}}_{m,i}}{dp_{i}}\right],$$

(see the electronic supplementary material). SA selection is expected to maintain genetic variation (i.e. conditions for balancing selection are met) when the strength of selection for a given locus is relatively symmetric between the sexes, in which case the terms in the square brackets of equations (2.6*a*) and (2.6*b*) must sum to zero (i.e. ${\bar{w}}_{\;f,i}^{-1}d{\bar{w}}_{\;f,i}/dp_{i}\approx -{\bar{w}}_{m,i}^{-1}d{\bar{w}}_{m,i}/dp_{i}$). Note that this condition applies whether or not there are dominance interactions, including dominance reversals [24,52–54], between SA alleles at a given locus. Substituting this identity into equations (2.5*a*) and (2.5*b*), we obtain:

2.7a $$f_{X}^{\mathrm{sel}}\approx \frac{9}{4}\overset{n_{X}}{\underset{i=1}{\sum }}\frac{{\hat{\;p}}_{i}(1-{\hat{\;p}}_{i})}{4n_{X}}{\left(\frac{1}{{\bar{w}}_{\;f,i}}\frac{d{\bar{w}}_{\;f,i}}{d{\hat{\;p}}_{\;f,i}}\right)}^{2}$$

and

2.7b $$f_{A}^{\mathrm{sel}}\approx \overset{n_{A}}{\underset{i=1}{\sum }}\frac{{\hat{\;p}}_{i}(1-{\hat{\;p}}_{i})}{4n_{A}}{\left(\frac{1}{{\bar{w}}_{\;f,i}}\frac{d{\bar{w}}_{\;f,i}}{d{\hat{\;p}}_{\;f,i}}\right)}^{2}.$$

With no systematic differences between the X and autosomes in the density of polymorphic SA loci, or the frequencies and fitness effects of their alleles, then the summation terms in $f_{X}^{\mathrm{sel}}$ and $f_{A}^{\mathrm{sel}}$ should have the same magnitudes, and the X-to-autosome ratio for the expected amount of *F*_ST_ inflation will be

2.8 $$\frac{\;f_{X}^{\mathrm{sel}}}{\;f_{A}^{\mathrm{sel}}}=\frac{9}{4}.$$

In other words, we expect a 2.25-fold higher inflation of ${\hat{F}}_{\mathrm{ST}}$ for the X compared to the autosomes *in the absence of chromosomal differences for SA polymorphisms*. This additional inflation for X-linked loci arises because haploidy in males inflates the contributions of X-linked loci to the variance for male fitness components (relative to autosomal loci) [23].

In practice, loci contributing to an inflation of *F*_ST_ may either be targets of selection or in linkage disequilibrium (LD) with such targets. While the prediction in equation (2.8) applies to targets of SA selection, it will remain applicable to linked loci provided their degree of LD with target loci does not systematically differ between the X chromosome and autosomes (see the electronic supplementary material). In our comparisons of the two chromosome types (see below), we used LD pruning to minimize the differential effects of hitchhiking between chromosomes. Because LD tends to be higher on the human X chromosome compared to autosomes [55], any uncontrolled chromosomal bias in the effects of LD on *F*_ST_ inflation should tend to increase the likelihood of identifying a signal of enriched SA polymorphism on the X, which makes our eventual conclusion (that there is no such signal) a conservative one.

Below, we empirically estimate the amount of inflation in ${\hat{F}}_{\mathrm{ST}}$ for the X and the autosomes in the UK Biobank (i.e. inflation relative to the average amount of noise in *F*_ST_ estimates, per chromosome), and compare these estimates to the baseline prediction of 9/4.

## 3. Methods

### (a) Quality-filtering of UK Biobank data

Access to UK Biobank data was granted under project number 52 049. We employed the same quality-filtering settings for X-linked loci in this study as employed for autosomal loci by Ruzicka *et al*. [48]. Briefly, we excluded individuals with high relatedness (3rd degree or closer), non-White British ancestry, high heterozygosity and missing rates, individuals whose reported sex and genetic sex differed, individuals whose age was less than 45 years, and aneuploids. Across retained individuals, we excluded non-diallelic sites, sites with MAF < 0.01, missing rates greater than 5%, *p*-values < 10^−6^ in tests of Hardy–Weinberg equilibrium, and imputation INFO score less than or equal to 0.8. Because there are few genotyped sites on the X chromosome, our analyses are focused on imputed data. Note that, unlike Ruzicka *et al*. [48], we did not filter our data for possible artefacts arising from mis-mapping of sequence reads to sex chromosomes [35,56] because reproductive *F*_ST_ controls for allele frequency differences between adults and, in effect, for artefacts arising when estimating adult allele frequencies.

### (b) Quantifying polygenic signals of sexually antagonistic polymorphism

For each X-linked locus, we estimated allele frequencies in adults (${\hat{\;p}}_{m}$ and ${\hat{\;p}}_{f}$). To quantify LRS, we used reported offspring numbers from UK Biobank field 2405 ‘Number of children fathered’ for males, and field 2743 ‘Number of live births' for females (see [48] for further details on quality-filtering). These data were used to estimate allele frequency transmission (${\hat{\;p}}_{m}^{\prime }$ and ${\hat{\;p}}_{f}^{\prime }$; equation (2.1)) and reproductive ${\hat{F}}_{\mathrm{ST}}$ for each polymorphic locus (equation (2.2)).

We generated a null distribution for reproductive ${\hat{F}}_{\mathrm{ST}}$ by simulating loci from the theoretical null model (i.e. equation (2.3*a*)), with the sample sizes per locus (*N_f_* and *N_m_* per locus) matching those in the UK Biobank data, and estimates of the sex-specific mean and variance for LRS based on all individuals included in the dataset. We also generated an empirical null distribution for ${\hat{F}}_{\mathrm{ST}}$ through a single permutation of LRS values (without permuting sex) and re-calculating ${\hat{F}}_{\mathrm{ST}}$ for each locus using permuted data. This permutation procedure ensured that neither sex differences in the allele frequencies among adults, nor sex differences in the relationship between genotype and LRS, contribute to ${\hat{F}}_{\mathrm{ST}}$ in the permuted data, leaving only estimation error (including error arising from locus-specific heterogeneity in LRS values owing to variation in missing rates across single nucleotide polymorphisms (SNPs)) to contribute to ${\hat{F}}_{\mathrm{ST}}$ in permuted data. We chose to perform a single permutation of LRS values for computational efficiency and because our focus was on testing significance across the set of loci, rather than establishing statistical significance for individual loci. We tested whether the distribution of ${\hat{F}}_{\mathrm{ST}}$ in observed data differed from both null distributions using Wilcoxon sum-rank and Kolmogorov–Smirnov tests.

To consolidate our inference that ${\hat{F}}_{\mathrm{ST}}$ inflations reflect genuine phenotypic effects (as opposed to technical artefacts), we assessed whether sites with elevated ${\hat{F}}_{\mathrm{ST}}$ were more likely to be functional. We first obtained variant effect predictions for each SNP using annotations from the hg19 reference genome in SNPEff [57], with sites categorized as ‘intergenic’ or ‘genic’, the latter defined broadly to include coding and potential regulatory genomic regions. We then performed logistic regressions, in which genic/intergenic was the binary response variable and ${\hat{F}}_{\mathrm{ST}}$ the independent variable, to assess relationships between ${\hat{F}}_{\mathrm{ST}}$ and genic/intergenic status. We also assessed whether associations with genic status were greater in observed than null (both simulated or permuted) data by re-calculating the regression coefficient of ${\hat{F}}_{\mathrm{ST}}$ on genic status among 1000 bootstrap replicates of the data, where one replicate consists of the set of SNPs sampled with replacement.

### (c) Comparing signals of sexually antagonistic polymorphism on autosomes and the X

We compared ${\hat{F}}_{\mathrm{ST}}$ on autosomes to ${\hat{F}}_{\mathrm{ST}}$ on the X chromosome by first estimating the ratio of mean ${\hat{F}}_{\mathrm{ST}}$ on the X relative to the autosomes (for simulated, permuted and observed data). We also estimated $f_{X}^{\mathrm{sel}}$ and $f_{A}^{\mathrm{sel}}$—each calculated as the difference between the observed mean ${\hat{F}}_{\mathrm{ST}}$ and the mean ${\hat{F}}_{\mathrm{ST}}$ from either the simulated or permuted null data—and the resulting $f_{X}^{\mathrm{sel}}$ to $f_{A}^{\mathrm{sel}}$ ratio. We obtained confidence intervals and empirical *p*-values for these ratios by sampling autosomal and X-linked loci with replacement, estimating the ratios in the resampled data and repeating this procedure across 1000 bootstrap replicates. In comparisons of autosomal and X-linked data, we focus on a set of LD-pruned loci (PLINK settings ‘–indep-pairwise 50 10 0.2’) to avoid biases arising from differences in the intensity of linked selection for autosomal and X-linked loci (see above).

## 4. Results

### (a) Signals of sexually antagonistic polymorphism for X-linked loci

**Figure 1.**
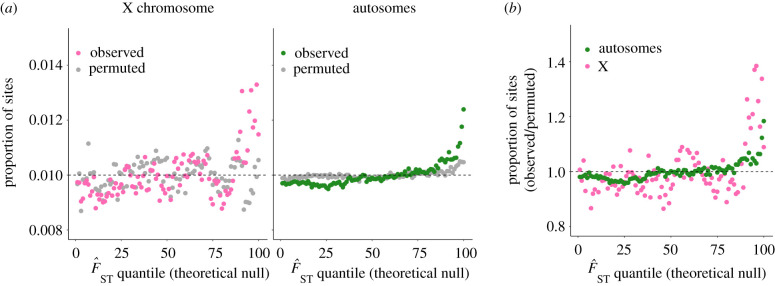
Polygenic signals of SA polymorphism on the X chromosome and autosomes relative to ‘no sex-differential selection’ nulls. (*a*) Proportion of X-linked sites (grey, permuted; pink, observed) and autosomal sites (grey, permuted; green, observed) in each of 100 quantiles of a simulated null distribution for ${\hat{F}}_{\mathrm{ST}}$, with the null distributions described by equations (2.3*a*) and (2.3*b*), respectively. In the absence of sex-differential selection, approximately 1% of sites should fall into each quantile of the simulated null distribution. (*b*) Observed/permuted ratio of the proportion of sites in each of 100 quantiles of the simulated null distribution, for X-linked (pink) and autosomal (green) sites, respectively. Both the above panels used the full set of *N_X_* = 229 196 and *N*_Auto._ = 7 851 642 imputed sites because: (i) signals of SA polymorphism relative to null distributions cannot be artificially inflated by LD between sites (when considering X-linked and autosomal sites separately; see [48]); and (ii) power to detect X versus autosome differences is reduced by LD pruning. We present equivalent figures for LD-pruned data as electronic supplementary material, figure S7. (Online version in colour.)

The total sample size, after quality-filtering, includes 249 021 individuals (*N_m_* = 115 531 and *N_f_* = 133 490), *N* = 229 196 imputed polymorphic sites on the X chromosome and *N* = 7 851 642 imputed polymorphic sites on autosomes. We first compared reproductive ${\hat{F}}_{\mathrm{ST}}$ on the X chromosome to a distribution of ${\hat{F}}_{\mathrm{ST}}$ simulated from the theoretical null distribution (based on equation (2.3*a*)) and to an empirical null distribution obtained by permuting LRS values within each sex (see Methods). As shown for autosomal loci previously (figure 1; [48]) and as predicted under SA selection, mean ${\hat{F}}_{\mathrm{ST}}$ for X-linked loci was elevated relative to both theoretical and empirical null distributions (theoretical $\bar{{\hat{F}}_{\mathrm{ST}}^{X}}=1.369\times 10^{-6}$; permuted; permuted ${\hat{F}}_{\mathrm{ST}}=1.365\times 10^{-6}$ observed $\bar{{\hat{F}}_{\mathrm{ST}}^{X}}=1.482\times 10^{-6}$; Wilcoxon rank-sum and Kolmogorov–Smirnov tests, all *p* < 0.001; figure 1; electronic supplementary material, figure S7). We observed a 14.8% enrichment of observed sites in the top 1% quantile of the theoretical null distribution (expected number of sites = 2292; observed number of sites = 2632; *χ*^2^ = 23.592, *p* < 0.001; figure 1; electronic supplementary material, figure S7) and a 8.4% enrichment of observed sites in the top 1% quantile of the empirical null distribution (expected number of sites = 2292; observed number of sites = 2484; *χ*^2^ = 7.719, *p* = 0.005). There were no individual large-effect loci contributing to this signal: the minimum *χ*^2^ *p*-value across all sites (1.213 × 10^−5^) was well above the Bonferroni-corrected threshold (2.182 × 10^−7^) and the minimum false dicovery rate *q*-value was 0.807. Thus, genomic signals of SA polymorphism for X-linked loci are polygenic.

If genic sites (broadly defined to include coding and regulatory regions) are more likely to have phenotypic effects than intergenic sites, and if ${\hat{F}}_{\mathrm{ST}}$ inflations reflect genuine phenotypic effects of SA polymorphisms, we should observe that sites with high ${\hat{F}}_{\mathrm{ST}}$ are more likely to be genic. As found for autosomes previously [48], we detected a positive association between ${\hat{F}}_{\mathrm{ST}}$ and genic status among X-linked polymorphisms (SNP coded as genic based on genome annotations; see Methods; binomial generalized linear model (GLM), log odds ratio (logOR) ± 95 confidence interval (CI) = 16 399.81[12 189.41–20 601.39], *p* < 0.001; electronic supplementary material, figure S8). By contrast, we found no significant association between permuted ${\hat{F}}_{\mathrm{ST}}$ and genic status (binomial GLM, logOR ± 95 CI = 3822.99[–679.01–8312.16], *p* = 0.096; electronic supplementary material, figure S8), or between simulated ${\hat{F}}_{\mathrm{ST}}$ and genic status (binomial GLM, logOR ± 95 CI = 2107.52[–2408.14–6610.39], *p* = 0.360), and the association between ${\hat{F}}_{\mathrm{ST}}$ and genic status was stronger in the observed than permuted data (1000 bootstrap replicates of the difference between the log odds-ratio in observed and permuted data = 12 680.27[6417–18 382.84], empirical *p* < 0.001). While these associations represent suggestive evidence ${\hat{F}}_{\mathrm{ST}}$ inflations are attributable to genuine phenotypic effects (and align with previous enrichment of candidate SA sites in functional genomic regions; e.g. [47]), we emphasize that the evidence is not definitive, as non-genic sites may be functional as well.

### (b) No evidence that sexually antagonistic polymorphisms are enriched on either chromosome type

Evidence for elevated reproductive ${\hat{F}}_{\mathrm{ST}}$ among X-linked loci (relative to the ‘no sex-differential selection’ null described by equation (2.3*a*)), together with previous evidence for elevated reproductive ${\hat{F}}_{\mathrm{ST}}$ on autosomes [48] (relative to the ‘no sex-differential selection’ null described by equation (2.3*b*)), suggest that SA polymorphisms segregate on both chromosome types. These findings provided motivation to compare signals of SA polymorphism on autosomes *relative to* the X.

**Figure 2.**
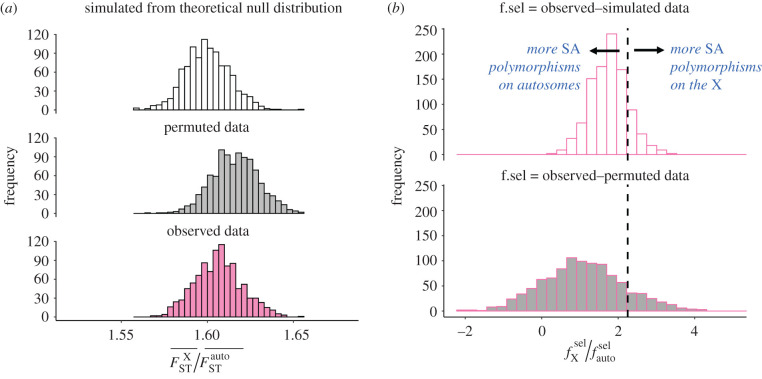
Comparing polygenic signals of SA polymorphism on the X chromosome and autosomes. (*a*) Distribution of the ratio of mean ${\hat{F}}_{\mathrm{ST}}$ ($\bar{{\hat{F}}_{\mathrm{ST}}^{X}}/\bar{{\hat{F}}_{\mathrm{ST}}^{A}}$) based on 1000 bootstrap replicates for each of our three classes of data: simulated data from the theoretical null distribution (top), permuted data (middle) and observed data (bottom), illustrating the elevation in X-linked relative to autosomal ${\hat{F}}_{\mathrm{ST}}$ (i.e. $\bar{{\hat{F}}_{\mathrm{ST}}^{X}}/\bar{{\hat{F}}_{\mathrm{ST}}^{A}}\gg 1$), even in the absence of SA polymorphism (i.e. in simulated and permuted data). (*b*) Distribution of the ratio of estimated X-linked to autosomal *inflation in* ${\hat{F}}_{\mathrm{ST}}$ ($f_{X}^{\mathrm{sel}}$ and $f_{A}^{\mathrm{sel}}$), across 1000 bootstrap replicates. The top panel uses observed and simulated data to estimate $f_{X}^{\mathrm{sel}}$ and $f_{A}^{\mathrm{sel}}$, while the bottom panel uses observed and permuted data to estimate $f_{X}^{\mathrm{sel}}$ and $f_{A}^{\mathrm{sel}}$. The dashed vertical line shows the theoretically predicted 9/4 X-to-autosome ratio when randomly distributed balanced SA polymorphisms account for the inflations of *F*_ST_ on each chromosome type. In both panels, we used a set of LD-pruned sites, rather than the full data, to avoid biases arising from differences in the extent of hitchhiking between autosomes and the X (see Methods). (Online version in colour.)

We first compared mean ${\hat{F}}_{\mathrm{ST}}$ between the X chromosome and autosomes (using a set of LD-pruned sites; *N_X_* = 29 859; *N*_Auto._ = 1 056 003), which should be larger for X-linked than autosomal loci (even in the absence of any SA polymorphism) because of the smaller sample sizes, and thus higher sampling variances, for X-linked loci. Accordingly, the ratio of mean X-linked to autosomal ${\hat{F}}_{\mathrm{ST}}$ (±95 CI) was greater than one (figure 2*a*), whether in simulated ${\hat{F}}_{\mathrm{ST}}$ values based on theoretical null distributions ($\bar{{\hat{F}}_{\mathrm{ST}}^{X}}=1.386\times 10^{-6}$, $\bar{{\hat{F}}_{\mathrm{ST}}^{A}}=8.661\times 10^{-7}$, $\bar{{\hat{F}}_{\mathrm{ST}}^{X}}/\bar{{\hat{F}}_{\mathrm{ST}}^{A}}=1.599[1.575\text{–}1.625]$), permuted data ($\bar{{\hat{F}}_{\mathrm{ST}}^{X}}=1.431\times 10^{-6}$, $\bar{{\hat{F}}_{\mathrm{ST}}^{A}}=8.853\times 10^{-7}$, $\bar{{\hat{F}}_{\mathrm{ST}}^{X}}/\bar{{\hat{F}}_{\mathrm{ST}}^{A}}=$ 1.616[1.589–1.643]), or observed data ($\bar{{\hat{F}}_{\mathrm{ST}}^{X}}=1.448\times 10^{-6}$, $\bar{{\hat{F}}_{\mathrm{ST}}^{A}}=9.016\times 10^{-7}$, $\bar{{\hat{F}}_{\mathrm{ST}}^{X}}/\bar{{\hat{F}}_{\mathrm{ST}}^{A}}=1.607[1.581\text{–}1.634]$).

To assess whether SA polymorphisms are enriched on autosomes or the X, we estimated, for each chromosome type, the degree of ${\hat{F}}_{\mathrm{ST}}$ inflation relative to the null (i.e. $f_{X}^{\mathrm{sel}}$ and $f_{A}^{\mathrm{sel}}$; see Theoretical Background) as the mean observed ${\hat{F}}_{\mathrm{ST}}$ minus the fraction of ${\hat{F}}_{\mathrm{ST}}$ that is attributable to sampling variance (i.e. mean simulated or permuted ${\hat{F}}_{\mathrm{ST}}$). We then compared the ratio of X to autosome ${\hat{F}}_{\mathrm{ST}}$ inflation to a baseline model in which the chromosome types exhibit no differences in SA polymorphism (i.e. $f_{X}^{\mathrm{sel}}/f_{A}^{\mathrm{sel}}=9/4=2.25$; equation (2.8)). We estimated $f_{X}^{\mathrm{sel}}/f_{A}^{\mathrm{sel}}$ as 1.770[0.828–3.290] based on observed relative to simulated ${\hat{F}}_{\mathrm{ST}}$ values, and $f_{X}^{\mathrm{sel}}/f_{A}^{\mathrm{sel}}$ as 1.086[–0.877–3.291] based on observed relative to permuted ${\hat{F}}_{\mathrm{ST}}$ values. Whereas enrichment of SA polymorphisms on the X would predict a ratio greater than 2.25, both point estimates fell below 2.25, though neither difference was statistically significant (empirical *p* = 0.853 and *p* = 0.864 for simulated and permuted data, respectively). Overall, there is no compelling evidence for an enrichment of SA polymorphisms on either chromosome type.

## 5. Discussion

Research on the genetic basis of SA fitness variation is motivated, in part, by its relevance to broader questions about adaptive evolution, such as the distribution of dominance coefficients among adaptive mutations [1,24,58], the extent of balancing selection in genomes [3] and the evolution of sex chromosome systems [59–62]. For example, evidence for enrichment of SA variants on the X chromosome might imply that SA polymorphisms evolve under balancing selection, with male-beneficial variants typically recessive and female-beneficial variants typically dominant [18,19]. On the other hand, evidence for enrichment of SA variants on autosomes might imply that SA variants exhibit co-dominant fitness effects [17], experience beneficial reversals of dominance [20] and/or have evolutionary dynamics dominated by directional selection or drift [26,27]. Yet empirically assessing whether SA polymorphisms are typically X-linked or autosomal is extremely challenging [23,34,35].

Here, we overcome the logistical hurdle of detecting SA polymorphisms by using a study organism—humans—in which there is previous evidence for SA selection [10,63,64] and a study population—the UK Biobank—in which sample sizes are large enough to detect polygenic signals of SA polymorphism [48]. We overcome the bias towards elevated X-linked between-sex allele frequency differentiation among adults [23,29,30] by using a metric of SA polymorphism—reproductive *F*_ST_—that controls for allele frequency differences among adults [48]. And we overcome the biases arising from larger effects of X-linked alleles on metrics of SA variation, owing to elevated X-linked sampling variances and X-linked haploidy in males [23,36], by developing models for reproductive *F*_ST_ estimates that account for these effects.

Our main finding was that X-linked sites displayed ${\hat{F}}_{\mathrm{ST}}$ inflations consistent with polygenic SA selection (as shown for autosomal sites previously [48]) but the extent of ${\hat{F}}_{\mathrm{ST}}$ inflation did not significantly differ from the 9/4 X-autosome ratio predicted by an idealized model in which SA polymorphisms are evenly distributed across the genome. These results accord well with re-analyses of previous empirical research in a range of species [23], which reveal little compelling evidence for enrichment of SA polymorphisms on the X chromosome once biases towards enhanced X-linked effects are accounted for. Indeed, enrichment of SA polymorphisms on the X chromosome might be considered unlikely for additional reasons. In particular, SA variants are likely to exhibit small fitness effects owing to the polygenic nature of fitness variation [28,65,66], as also evidenced by the absence of large-effect SA loci in this dataset. Small-effect mutations, including SA mutations, are highly susceptible to genetic drift and potentially more likely to be co-dominant [66,67]—conditions that both favour autosomal enrichment of SA polymorphisms [17,26,27]. Furthermore, fitness effects of SA variants may be asymmetric between sexes owing to well-documented sex differences in strategies employed to achieve reproductive success [8,68–70]. This could render SA variants susceptible to directional (rather than balancing) selection, which also tends to favour autosomal enrichment of SA polymorphisms [26,27].

### (a) Limitations of our analysis approach and future directions

Though our analyses correct for several pre-existing biases, they present some limitations. In terms of the data, the relatively small number of independently segregating polymorphisms across the human X chromosome (*N* ∼ 30 000 LD-pruned imputed sites) reduces power to detect differences between autosomal and X-linked sites. While there is prior support for SA selection on phenotypes [64] and for polygenic signals of autosomal SA polymorphism [48] in this population, the extent to which ${\hat{F}}_{\mathrm{ST}}$ inflations are driven by SA polymorphisms, as opposed to some loci showing sex differences in directional selection and neutral sites linked to selected polymorphisms, is unclear. Empirical work in systems where fitness can be measured relatively easily, sufficiently large samples of genomic sequences can be obtained, and experimental work can be carried out to test the fitness effects of candidate SA polymorphisms (e.g. the dioecious plant *Silene latifolia*; [9]), represent promising avenues for further research. Although there is little prospect of obtaining Biobank-scale samples of genome sequences in non-human organisms, we suspect that genetic variation for fitness may often be greater than in humans, increasing the likelihood that polygenic signals of SA selection will be detectable [16,48].

Regarding limitations of theory, previous research (including our own) has predominantly used single-locus models to make predictions about the genomic distribution of SA polymorphisms [17–23,27,71], despite the (likely) polygenic nature of fitness variation. Though we can extend single-locus predictions to polygenic scenarios when we assume that loci segregate independently and fitness effects are multiplicative, this simplifying assumption may be problematic when the extent of linked selection differs systematically between autosomal and X-linked loci [25]. Moreover, the 9/4 ratio of ${\hat{F}}_{\mathrm{ST}}$ inflation is a prediction for signals of SA polymorphism assuming a random genomic distribution of *balanced* SA polymorphisms, yet balancing selection is only one of the multiple modes of evolution that can potentially affect SA polymorphisms [26,27,65]. Empirical data from the UK Biobank are consistent with the 9/4 prediction, yet our failure to reject this model should not be interpreted as evidence that the evolutionary scenario underlying this prediction is a reasonable description of genome-wide SA polymorphism in humans (or other species). Other evolutionary scenarios (e.g. interactions between recurrent mutation, genetic drift, and the distributions of sex-specific selection and dominance coefficients) might also lead to predictions close to 9/4. Finally, most current theoretical models of SA variation fall firmly within the classical population genetic tradition [72], in which the fitness effects of SA variants are arbitrarily assigned rather than explicitly modelled. Because fitness effects of genetic variation are properties of the distribution of mutations affecting traits under selection, models of adaptation that incorporate these features of biology (e.g. Fisher's Geometric model [4,58] and other trait-based population genetic models [65]) may bring us closer to a robust theory for the fitness effects, dynamical properties and genomic distribution of SA polymorphisms.

## Data accessibility

Code is available from GitHub (/filipluca/Polygenic_SA_selection_X_vs_A), along with metadata (within the restrictions of the UK Biobank agreement) from the Dryad Digital Repository (https://doi.org/10.5061/dryad.pnvx0k6p6) [73] and additional data in the electronic supplementary material [74].
